# Co-infection of porcine deltacoronavirus and porcine epidemic diarrhea virus induces early TRAF6-mediated NF-κB and IRF7 signaling pathways through TLRs

**DOI:** 10.1038/s41598-022-24190-w

**Published:** 2022-11-14

**Authors:** Kepalee Saeng-chuto, Adthakorn Madapong, Kampon Kaeoket, Pablo Enrique Piñeyro, Angkana Tantituvanont, Dachrit Nilubol

**Affiliations:** 1 Swine Viral Evolution and Vaccine Development Research Unit, Department of Veterinary Microbiology, Faculty of Veterinary Science, Chulalongkorn University, Henry Dunant Road, Pathumwan, 10330 Bangkok Thailand; 2 Department of Clinical Sciences and Public Health, Faculty of Veterinary Science, Mahidol University, Nakhon Pathom, Thailand; 3 Department of Veterinary Diagnostic and Production Animal Medicine, College of Veterinary Medicine, Iowa State University, Ames, IA USA; 4 Department of Pharmaceutics and Industrial Pharmacy, Faculty of Pharmaceutical Sciences, Chulalongkorn University, Bangkok, Thailand; 5 Cell-Based Drug and Health Product Development Research Unit, Faculty of Pharmaceutical Sciences, Chulalongkorn University, Bangkok, Thailand

**Keywords:** Immunology, Microbiology

## Abstract

Porcine deltacoronavirus (PDCoV) and porcine epidemic diarrhea virus (PEDV) infect the small intestine and cause swine enteric coronavirus disease. The mucosal innate immune system is the first line of defense against viral infection. The modulatory effect of PDCoV and PEDV coinfection on antiviral signaling cascades of the intestinal mucosa has not been reported. Here, we investigate the gene expression levels of pattern recognition receptors, downstream inflammatory signaling pathway molecules, and associated cytokines on the intestinal mucosa of neonatal piglets either infected with a single- or co-infected with PDCoV and PEDV using real-time PCR. The results demonstrate that single-PEDV regulates the noncanonical NF-κB signaling pathway through RIG-I regulation. In contrast, single-PDCoV and PDCoV/PEDV coinfection regulate proinflammatory and regulatory cytokines through TRAF6-mediated canonical NF-κB and IRF7 signaling pathways through TLRs. Although PDCoV/PEDV coinfection demonstrated an earlier modulatory effect in these signaling pathways, the regulation of proinflammatory and regulatory cytokines was observed simultaneously during single viral infection. These results suggested that PDCoV/PEDV coinfection may have synergistic effects that lead to enhanced viral evasion of the mucosal innate immune response.

## Introduction

Porcine epidemic diarrhea virus (PEDV) and porcine deltacoronavirus (PDCoV) are enveloped, positive-sense, single-stranded RNA (ssRNA) viruses belonging to the family *Coronaviridae*, order *Nidovirales*^1^, and genera *Alphacoronavirus* and *Deltacoronavirus*, respectively^1^. The PEDV and PDCoV genomes are approximately 28 and 25 kb in length, respectively, and their genome organizations differ slightly. The PEDV genome contains seven open reading frames (ORF): ORF 1a and 1b, spike (S), envelope (E), membrane (M), and nucleocapsid (N) genes, and ORF3 and is flanked by a 5′- and 3′-untranslated region (UTR). In contrast, non-structural protein 6 (Nsp6) and Nsp7 are found in PDCoV genome^2,3^. Porcine epidemic diarrhea virus and PDCoV can coinfect porcine small intestinal enterocytes, leading to a severe enteric disease collectively named swine enteric coronavirus-disease (SECD). The disease is characterized by malabsorption, diarrhea, dehydration, vomiting, and high mortality, especially in neonatal piglets^4,5^. The severity of SECD depends on the age of the infected pigs and the virus strain^6–8^. Thus, PEDV and PDCoV result in serious economic and production losses within the swine industry worldwide.

The innate immune system is the first line of defense against viral infection. Viral pathogen-associated molecular patterns (PAMPs) are signature molecules expressed by viruses and recognized by host pattern recognition receptors (PRRs) on innate immune cells, such as epithelial cells lining internal organs, dendritic cells (DC) and macrophages^9^. Pattern recognition receptors, such as retinoic acid-inducible gene I (RIG-I)-like receptors and members of toll-like receptor (TLRs) family, including TLR2, TLR3, TLR4, TLR7, TLR8, and TLR9, play a vital role in recognizing viruses, leading to the activation of antiviral signaling cascades^10–13^. Myeloid differentiation factor 88 (MyD88) and toll/interleukin-1 receptor-domain-containing adaptor-inducing interferon-β (TRIF) are two major downstream adaptors that facilitate TLR signaling. Meanwhile, RIG-I-like receptors elicits an antiviral response through the cellular adaptor, called mitochondrial antiviral signaling protein (MAVS). The activation of RIG-I and the TLRs’ downstream adaptors, MyD88 and TRIF, lead to the recruitment of tumor necrosis factor (TNF) and receptor-associated factor 6 (TRAF6), resulting in the activation of interferon regulatory factors (IRFs) and the nuclear factor kappa-light-chain-enhancer of activated B cells (NF-κB)^14,15^, which regulate the final production of type I interferon (IFN) and inflammatory cytokines^16^. Recently, many studies have reported that either PEDV or PDCoV single-infection activates antiviral signaling cascades and, more specifically, upregulates the expression of IFN-α and IL12 in either single infection or coinfection in the small intestinal mucosa of neonatal piglets^12,17,18^. The modulatory effect of the innate immune response and signaling cascade during PEDV and PDCoV coinfection differs from that observed in individual infection and seems to have detrimental synergism, impairing host intracellular processes, which results in a defective mucosal immune response. Therefore, in this study, we investigate the gene regulation of PRRs, downstream inflammatory signaling pathway molecules, and proinflammatory and regulatory cytokines on the small intestinal mucosa of neonatal piglets inoculated with PDCoV and/or PEDV.

## Materials and methods

### Virus isolates and propagation

The PDCoV isolate NT1_1215 (KX361345) and PEDV isolate P1915-NPF-071511A (KX981900) were used in this study. Virus propagation was completed on LLC-PK1 (ATCC® CL-101™) and Vero C1008 cells (ATCC® CRL-1586™) for PDCoV and PEDV, respectively. Briefly, LLC-PK1 and Vero C1008 cells were maintained on growth media (Dulbecco’s Modified Eagle Medium (DMEM; Gibco, USA) supplemented with 10% heat-inactivated fetal bovine serum (FBS; Gibco, USA). When the cells reached approximately 80% confluency, they were washed twice with 1X phosphate-buffered saline (PBS; 0.1 M, pH 7.4) and placed in maintenance media (DMEM (Gibco, USA) supplemented with 8 µg/ml trypsin/EDTA (Gibco, USA). Each virus was inoculated in their respective cell line and incubated for 1 h at 37 °C and 5% CO_2_, followed by two washes with 1X PBS. After virus inoculation, the cells were incubated in maintenance media at 37 °C and 5% CO_2_ until a cytopathic effect (CPE) was observed.

### Ethical statement for experimental procedures

According to the protocols reviewed and approved by the Faculty of Veterinary Science, Mahidol University-Institute Animal Care and Use, all animal procedures were performed according to the Guide for the Care and Use of Laboratory Animals of the National Research Council Thailand Committee (FVS-MU-IACUC; animal use license number U1-01281-2558). This study is reported in accordance with ARRIVE guidelines.

### Experimental design

Twenty-four 4-day-old castrated piglets from a commercial farm with no history of PDCoV or PEDV were randomly allocated into four groups (n = 6 pigs per group) based on weight stratification. Piglets were maintained in piglet nursery crates and acclimatized for 24 h before orally inoculation. Piglets were clinically evaluated daily for the presence of vomiting, diarrhea, lethargy, dehydration, and body condition as previously described^18^. The four treatment groups included (G1) the single-PDCoV infected group (n = 6), (G2) the single-PEDV infected group (n = 6), (G3) the PDCoV/PEDV co-infected group (n = 6), and (G4) a negative control group (n = 6). Pigs in groups G1 and G2 were orally inoculated with 5 ml (10^3^ TCID_50_/ml) of PDCoV and PEDV, respectively. Pigs in group G3 were orally inoculated with 5 ml of a mixture of 2.5 ml of PDCoV and 2.5 ml of PEDV (10^3^ TCID_50_/ml). Pigs in group G4 were orally inoculated with 5 ml of media. Three pigs in each group were euthanized at 3 and 5 days post-inoculation (DPI) by an intravenous injection of sodium pentobarbital and then euthanized by overdose administration. A radom segment of jejunum was dissected and rinsed with 1X PBS, pH 7.4, to remove the intestinal contents. Five milligrams (mg) of intestinal mucosa were collected by scraping with a sterile scalpel blade and maintained in RNAlater™ Stabilization Solution (Life Technologies, Carlsbad, CA). All sows and piglets used in this study were confirmed negative for PDCoV, PEDV, transmissible gastroenteritis virus (TGEV), and porcine rotavirus by virus-specific polymerase chain reaction (PCR) on fecal swabs previous allocation into research facilities^19–22^.

After inoculation, fecal swabs were collected daily using individually sterile swabs and placed immediately on 1 ml of RNAlater™ (Thermo-Fisher Scientific, MA, USA). Fecal samples were evaluated for the presence of viral RNA to confirm either PDCoV or PEDV shedding status. Viral RNA was extracted from feces by using a Nucleospin® Viral RNA Extraction Kit (Macherey-Nagel Inc., PA, USA), following the manufacturer’s instruction, and then converted into cDNA using M-MuLV Reverse Transcriptase (New England BioLabs Inc., MA, USA). The cDNA was used for viral detection using PCR with specific PDCoV and PEDV primers previously described^20,21^. The PCR products were confirmed via gel electrophoresis in a 1% agarose gel (100 V for 30 min), followed by nucleic acid detection with RedSafe™ staining solution (iNtRON Biotechnology Inc., Seongnamsi, South Korea), and examined under UV light. After confirmation of the amplicon size, the PCR products were purified (Nucleospin® Gel) by using a PCR clean-up kit (Macherey–Nagel Inc., Bethlehem, PA, USA) and sequenced (First BASE Laboratories Inc. (Selangor, Malaysia).

### Expression of mRNA for PRRs, inflammatory signaling pathway molecules, cytokines, and chemokines in porcine small intestinal mucosa

Total RNA was extracted from five mg of intestinal mucosa using a Qiagen RNeasy® Plus mini kit (Qiagen; Hilden, Germany). Two micrograms (µg) of total RNA were converted into cDNA using M-MuLV Reverse Transcriptase (New England BioLabs Inc., MA, USA). The cDNA was used to measure the relative fold changes in the gene expression of PRRs (TLR2, TLR3, TLR4, TLR7, TLR8, TLR9, and RIG-1), downstream inflammatory signaling pathway molecules (TRIF, MyD88, TRAF6, IRF7, NF-κB1 (p105), NF-κB1 (p50), and RelA (p65)), and cytokines (IL1-α, IL1-β, IL6, IL8, IL10, IL18, IL22, IL23p19, IL33, and TNF-α) using qPCR with specific primers, as previously reported^12,23–25^. The qPCR reaction was performed using Maxima SYBR Green/ROX qPCR Master Mix (2X) (Thermo-Fisher Scientific, MA, USA) in the QuantStudio 3D Digital PCR System (Applied Biosystems, Waltham, MA, USA). Each sample was run in triplicate. The mRNA expression levels were evaluated using the 2^−ΔΔCt^ method previously reported^26^. Glyceraldehyde-3-phosphate dehydrogenase (GAPDH) and beta-actin were used as internal controls to normalize changes in specific gene expressions. The results were presented as fold changes relative to the control animals.

### Statistical analysis

Fold changes between groups were compared using a one-way analysis of variance, followed by Tukey’s multiple comparison test in GraphPad Prism 7 (GraphPad Software Inc., La Jolla, CA, USA). Significant changes were identified when the *p* value was < 0.05.

## Results

### PDCoV and PEDV co-infection have an early up-regulatory effect on TLRs but a down-regulatory effect on RIG-I gene expression

**Figure 1:**
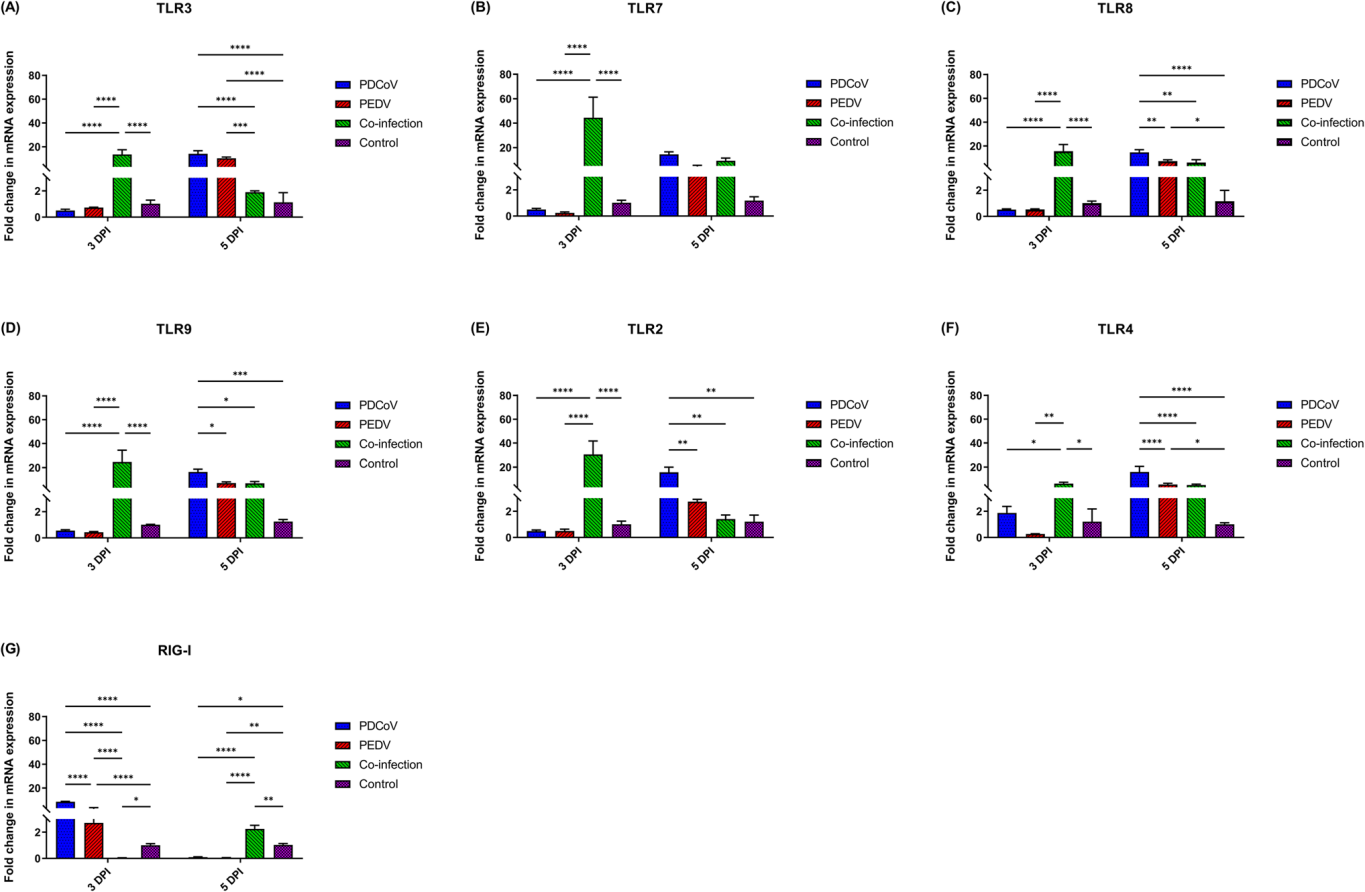
Messenger RNA (mRNA) expression of pattern recognition receptors (PRRs) including members of toll-like receptor (TLRs) family and retinoic acid-inducible gene I (RIG-I)-like receptors on the intestinal mucosa of neonatal pigs. (**A**) The mRNA levels of TLR3. (**B**) The mRNA levels of TLR7. (**C**) The mRNA levels of TLR8. (**D**) The mRNA levels of TLR9. (**E**) The mRNA levels of TLR2. (**F**) The mRNA levels of TLR4. (**G**) The mRNA levels of RIG-I.The mRNA levels were determined using qPCR. Glyceraldehyde-3-phosphate dehydrogenase (GAPDH) and beta-actin were used as an internal control to normalize changes in specific gene expressions. The results were presented as fold changes relative to the control animals. Different lower-case letters indicate significant differences between each group and each day post inoculation (DPI) (*p* < 0.05).

Host pattern recognition receptors (PRRs), such as TLRs and RIG-I-like receptors, play an essential role in viral recognition and the activation of the antiviral signaling cascade^10–13^. Neither the single-PDCoV- nor -PEDV-inoculated groups showed a significant mRNA regulatory effect on endosomal TLR3, TLR7, TLR8, and TLR9 genes at 3 DPI (Fig. 1A–D). However, at 5 DPI, the significant up-regulation of TLR3 (PDCoV: 14.01-fold changes; *p* < 0.0001; PEDV: 10.27-fold changes; *p* < 0.0001) and TLR8 (PDCoV: 14.62-fold changes; *p* < 0.0001; PEDV: 7.32-fold changes; *p* = 0.0231) was observed in both individually inoculated groups as compared with control animals, and a significantly higher TLR3 regulatory effect (14.01- versus 1.90-fold change; *p* < 0.0001) as compared with the PDCoV/PEDV-co-inoculated group was observed. At 5 DPI, the single-PDCoV group also showed the significant up-regulation of TLR9 as compared with animals in the control group (16.34-fold changes; *p*_=_ 0.0004). Moreover, the single-PDCoV group showed a significantly higher TLR8 and TLR9 regulatory effect as compared with the single-PEDV-inoculated group (TLR8: 14.62- versus 7.32-fold change; *p* = 0.0068; TLR9: 16.34- versus 7.01-fold change; *p* = 0.0241) or the PDCoV/PEDV-co-inoculated group (TLR8: 14.62- versus 6.09-fold change; *p* = 0.0018; TLR9: 16.34- versus 7.01-fold change; *p* = 0.0203) (Fig. 1A–D).

Neither pigs in the single-PDCoV group nor PEDV-infected animals showed significant changes in TLR2 (PDCoV and PEDV: *p* = 0.9988) or TLR4 (PDCoV: *p* = 0.9656; PEDV: *p* = 0.9130) mRNA expression levels at 3 DPI (Fig. 1E, F). However, the significant up-regulation of TLR2 (PDCoV: 4.33-fold changes; *p* = 0.0039) mRNA level was detected in the single-PDCoV group, and that of TLR4 (PDCoV: 16.01-fold changes; *p* < 0.0001; PEDV: 5.35-fold changes; *p* = 0.0367) in both single-infection groups was detected at 5 DPI as compared with control animals. Moreover, the single-PDCoV group showed significantly higher TLR2 and TLR4 mRNA levels than the single-PEDV group (TLR2: 15.68 versus 2.74-fold changes; *p* = 0.0094; TLR4: 16.01- versus 5.35-fold changes; *p* < 0.0001) or PDCoV/PEDV co-inoculated group (TLR2: 15.68 versus 1.41-fold changes; *p* = 0.0043; TLR4: 16.01- versus 4.93-fold changes; *p* < 0.0001) (Fig. 1E, F).

The RIG-I gene’s modulatory effect showed a variable pattern over time. RIG-I mRNA levels were significantly up-regulated at 3 DPI for animals in the single-PDCoV (8.52-fold changes; *p* < 0.0001) and -PEDV groups (2.71-fold changes; *p* = 0.0002) as compared with animals in the control group (Fig. 1G). However, both single inoculated groups showed a significant down-regulatory effect on RIG-I at 5 DPI (PDCoV: 0.10-fold changes; *p* = 0.0133; PEDV: 0.05-fold changes; *p* = 0.0091). Conversely, the PDCoV/PEDV-co-inoculated group showed a significant down-regulation of RIG-I mRNA levels at 3 DPI (0.04-fold changes; *p* = 0.0106) but significant up-regulation at 5 DPI (2.25-fold changes; *p* = 0.0014) as compared with animals in the control group (Fig. 1G).

### PDCoV and PEDV co-infection induce the early TRAF6-mediated canonical activation of NF-κB

**Figure 2:**
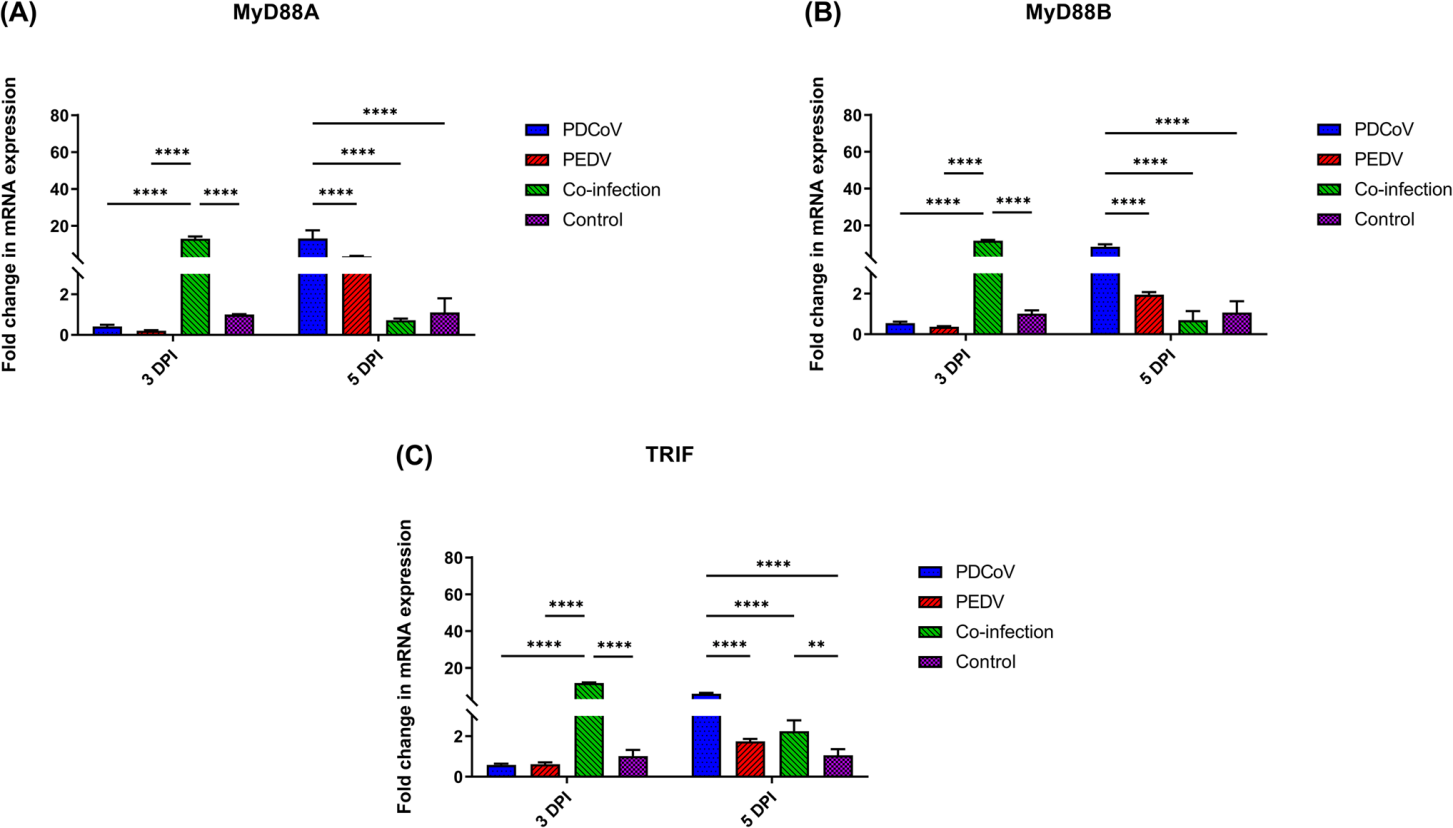
Messenger RNA (mRNA) expression of toll-like receptors (TLRs) associated with adaptor proteins on the intestinal mucosa of neonatal pigs. (**A**) The mRNA levels of myeloid differentiation factor 88 A (MyD88A). (**B**) The mRNA levels of myeloid differentiation factor 88 B (MyD88B). (**C**) The mRNA levels of toll/interleukin-1 receptor-domain-containing adaptor-inducing interferon-β (TRIF). The mRNA levels were determined using qPCR. Glyceraldehyde-3-phosphate dehydrogenase (GAPDH) and beta-actin were used as an internal control to normalize changes in specific gene expressions. The results were presented as fold changes relative to the control animals. Different lower-case letters indicate significant differences between each group and each day post inoculation (DPI) (*p* < 0.05).

MyD88 and TRIF are intracellular adaptor proteins that play an essential role in TLR signaling pathways and the activation of proinflammatory response. No differences in mRNA levels of MyD88A, MyD88B, and TRIF were observed for single-PDCoV-inoculated pigs as compare with the control group at 3 DPI; however, significant up-regulation was observed by 5 DPI (*p* < 0.0001) (Fig. 2). Single-PEDV inoculation did not affect MyD88 and TRIF mRNA levels at 3 or 5 DPI as compared to the control group (Fig. 2). At 3 DPI, the PDCoV/PEDV co-inoculation group showed significant up-regulation of MyD88A, MyD88B, and TRIF (13.96-, 11.73-, and 11.85-fold changes, respectively; *p* < 0.0001) (Fig. 2). However, by 5 DPI, only the mRNA levels of the TRIF gene in the PDCoV/PEDV co-inoculation group were up-regulated (2.25-fold change; *p* = 0.0018) as compared with animals in the control group (Fig. 2C).

**Figure 3:**
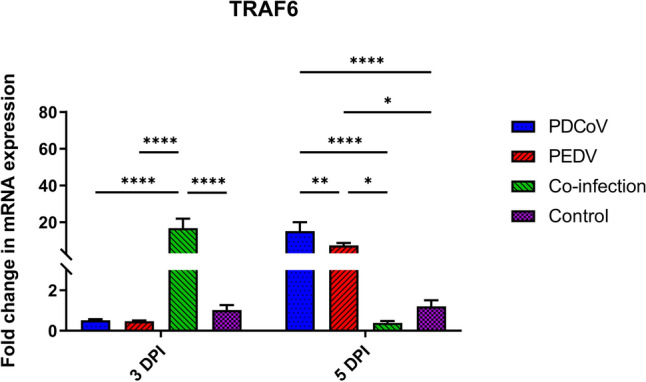
Messenger RNA (mRNA) expression of tumor necrosis factor (TNF) receptor-associated factor 6 (TRAF6) on the intestinal mucosa of neonatal pigs. The mRNA levels were determined using qPCR. Glyceraldehyde-3-phosphate dehydrogenase (GAPDH) and beta-actin were used as an internal control to normalize changes in specific gene expressions. The results were presented as fold changes relative to the control animals. Different lower-case letters indicate significant differences between each group and each day post inoculation (DPI) (*p* < 0.05).

**Figure 4:**
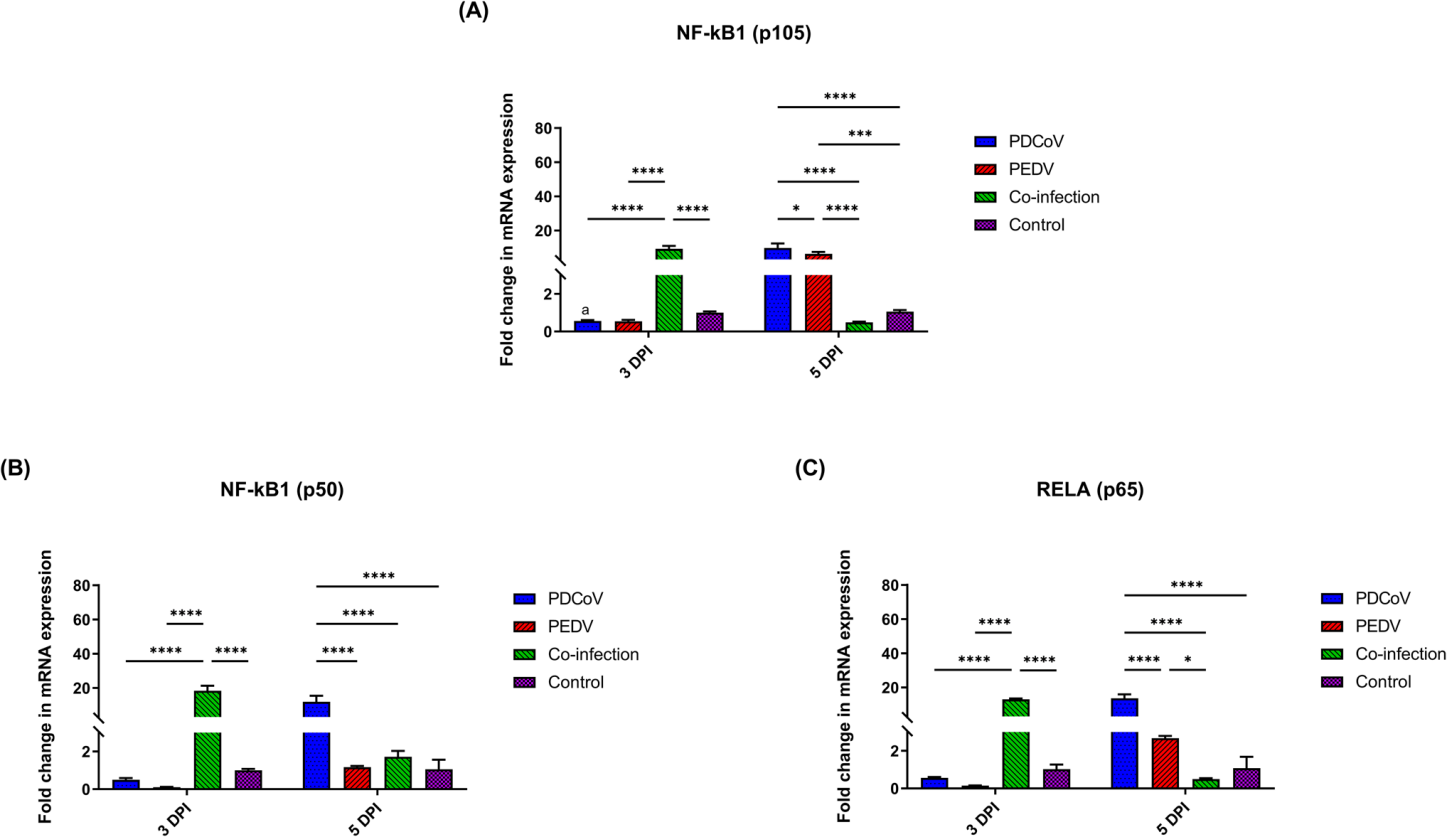
Messenger RNA (mRNA) expression of canonical nuclear factor kappa-light-chain-enhancer of activated B cells (NF-κB) family members on the intestinal mucosa of neonatal pigs. (**A**) The mRNA levels of NF-κB1 (p105). (**B**) The mRNA levels of NF-κB1 (p50). (**C**) The mRNA levels of RelA (p65). The mRNA levels were determined using qPCR. Glyceraldehyde-3-phosphate dehydrogenase (GAPDH) and beta-actin were used as an internal control to normalize changes in specific gene expressions. The results were presented as fold changes relative to the control animals. Different lower-case letters indicate significant differences between each group and each day post inoculation (DPI) (*p* < 0.05).

The activation of MyD88 and TRIF lead to the modulation of TRAF6, one of the essential adaptor proteins involved in the NF-κB signaling pathway. By 3 DPI, TRAF6 gene expression was only significantly up-regulated in the PDCoV/PEDV-co-inoculation group as compared to the control (16.78-fold changes; *p* < 0.0001) and single-inoculated groups (PDCoV: 0.52-fold changes; *p* < 0.0001; PEDV: 0.47-fold changes; *p* < 0.0001) (Fig. 3). However, the TRAF6 mRNA levels in the PDCoV/PEDV-co-inoculated group were comparable with those of the control group by 5 DPI. Conversely, the single-PDCoV and -PEDV groups showed the significant upregulation of the TRAF6 gene by 5 DPI (PDCoV: 15.12-fold changes; *p* < 0.0001; PEDV 7.37-fold changed; *p* = 0.0424). A similar regulatory pattern to that of TRAF6 was observed for NF-κB1 (p105), NF-κB1 (p50), and RelA (p65) gene expression. Compared with the control group, the PDCoV/PEDV co-inoculated group showed up regulatory effect on the part of the NF-κB1 (p105), NF-κB1 (p50), and RelA (p65) genes (9.31-, 18.42-, and 13.02-fold changes, respectively; *p* < 0.0001) by 3 DPI. However, mRNA levels at 5 DPI were comparable with the control group (Fig. 4). In the single-PDCoV group, there was the up-regulation of NF-κB1 (p105), NF-κB1 (p50), and RelA (p65) genes at 5 DPI (9.86-, 11.98-, and 13.60-fold changes, respectively; *p* < 0.0001) (Fig. 4), while in single-PEDV group, only the NF-κB1 (p105) gene was up-regulated at 5 DPI (6.38-fold changes; *p* = 0.0002) as compared to the control group (Fig. 4A).

### PDCoV and PEDV co-infection induce the early activation of IRF7

**Figure 5:**
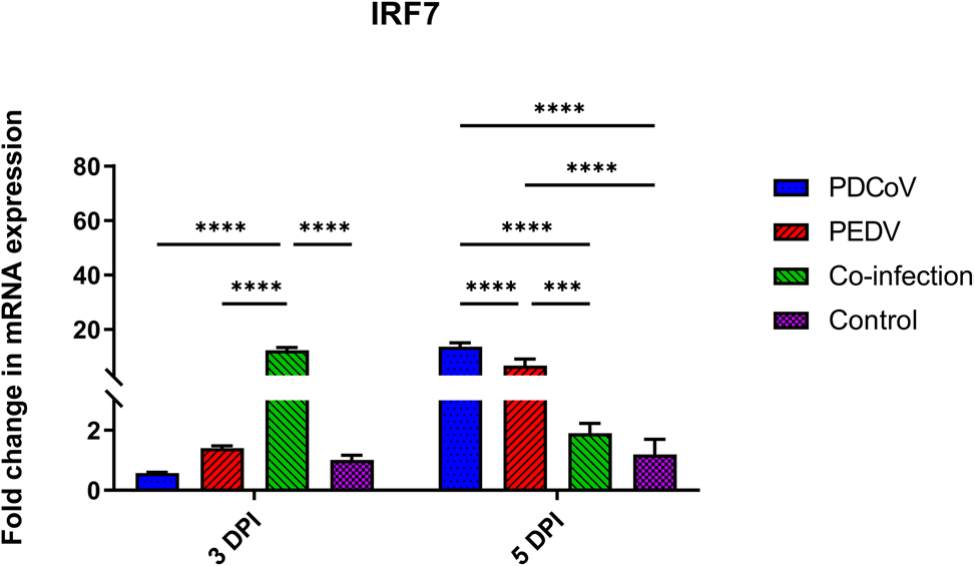
Messenger RNA (mRNA) expression of interferon regulatory factor 7 (IRF7) on the intestinal mucosa of neonatal pigs. The mRNA levels were determined using qPCR. Glyceraldehyde-3-phosphate dehydrogenase (GAPDH) and beta-actin were used as an internal control to normalize changes in specific gene expressions. The results were presented as fold changes relative to the control animals. Different lower-case letters indicate significant differences between each group and each day post inoculation (DPI) (*p* < 0.05).

Not only the canonical NF-κB pathway but also the IRFs pathway is activated by TRAF6. In fact, IRF7, a master regulator of type-I IFN production, did not show a significant modulatory effect on the single-PDCoV or -PEDV groups by 3 DPI; however, mRNA levels were significantly higher in both single-inoculation groups at 5 DPI as compared with the control group (PDCoV: 13.64-fold changes; *p* < 0.0001; PEDV: 6.73-fold changes; *p* < 0.0001) or the PDCoV/PEDV-co-inoculated group (PDCoV: 13.64- versus 1.90-fold change; *p* < 0.0001; PEDV: 6.73- versus 1.90-fold change; *p* = 0.0002) (Fig. 5). In contrast, the PDCoV/PEDV-co-inoculated group showed an early positive regulatory effect on IRF7 at 3 DPI (12.38-fold changes; *p* < 0.0001), but no modulatory effect was observed by 5 DPI (Fig. 5).

### Both single-PDCoV or -PEDV infection and PDCoV/ PEDV co-infection lead to cytokine storm at 5 days post infection

**Figure 6:**
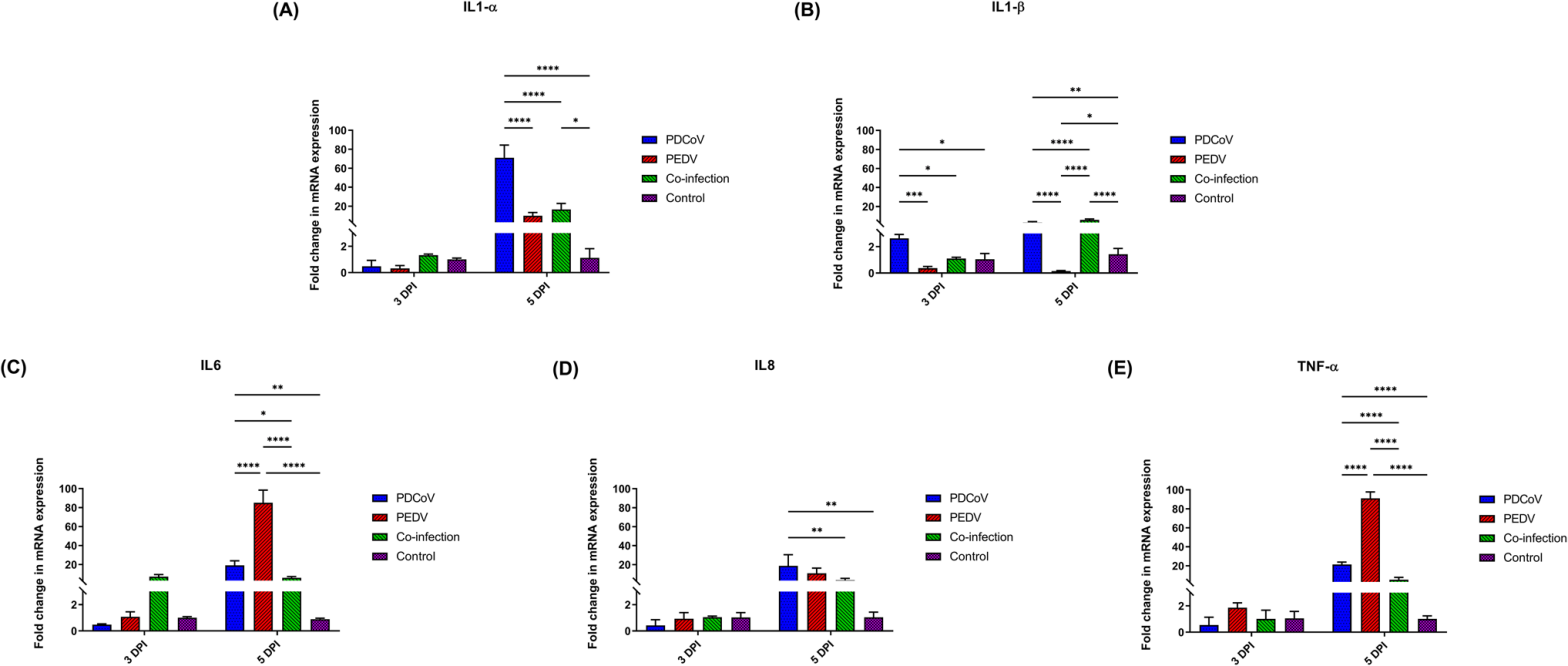
Messenger RNA (mRNA) expression of proinflammatory cytokines on the intestinal mucosa of neonatal pigs. (**A**) The mRNA levels of interleukin 1 alpha (IL1-α). (**B**) The mRNA levels of interleukin 1 beta (IL1-β). (**C**) The mRNA levels of interleukin 6 (IL6). (**D**) The mRNA levels of interleukin 8 (IL8). (**E**) The mRNA levels of tumor necrosis factor-alpha (TNF-α). The mRNA levels were determined using qPCR. Glyceraldehyde-3-phosphate dehydrogenase (GAPDH) and beta-actin were used as an internal control to normalize changes in specific gene expressions. The results were presented as fold changes relative to the control animals. Different lower-case letters indicate significant differences between each group and each day post inoculation (DPI) (*p* < 0.05).

The activation of TLRs and RIG-I lead to antiviral signaling cascade activation, resulting in the production of cytokines, which are critical to viral elimination^27,28^. The expression levels of proinflammatory cytokine genes, including IL1-α, IL1-β, IL6, IL8, and TNF-α, are presented in Fig. 6. Also, PDCoV/ PEDV co-inoculation led to the significant up-regulation of the IL1-α (16.50- fold changes; *p* = 0.0138) and IL1-β (5.88-fold changes; *p* < 0.0001) genes at 5 DPI as compared to the control group (Fig. 6A,B). The single-PDCoV inoculation group showed the significantly induced expression of IL1-β at 3 DPI (2.64-fold change; *p* = 0.0125) and a significantly higher IL1-β regulatory effect as compared with single-PEDV-inoculation group (2.64- versus 0.37-fold change; *p* = 0.0006) and the PDCoV/PEDV co-inoculated group (2.64- versus 1.11-fold change; *p* = 0.0164). Moreover, the single-PDCoV group showed a significantly up-regulatory effect on all proinflammatory cytokine genes, including the IL1-α, IL1-β, IL6, IL8, and TNF-α genes, at 5 DPI as compared to the control group (IL1-α: 71.09-fold changes; *p* < 0.0001; IL1-β: 3.16-fold changes; *p* = 0.0066; IL6: 19.06-fold changes; *p* = 0.0025; IL8:18.74-fold change; *p* = 0.0012; TNF-α: 21.42-fold changes; *p* < 0.0001) (Fig. 6). The single-PEDV inoculation group showed no effect on all proinflammatory cytokine genes at 3 DPI as compared to the control group. However, the single-PEDV group showed a significantly up-regulatory effect on IL6 and TNF-α (IL6: 85.11-fold changes; *p* < 0.0001; TNF-α: 90.98-fold changes; *p* < 0.0001) (Fig. 6C, E) and a down-regulatory effect on IL1-β (0.15-fold changes; *p* = 0.0494) at 5 DPI as compared to the control group (Fig. 6B).

**Figure 7:**
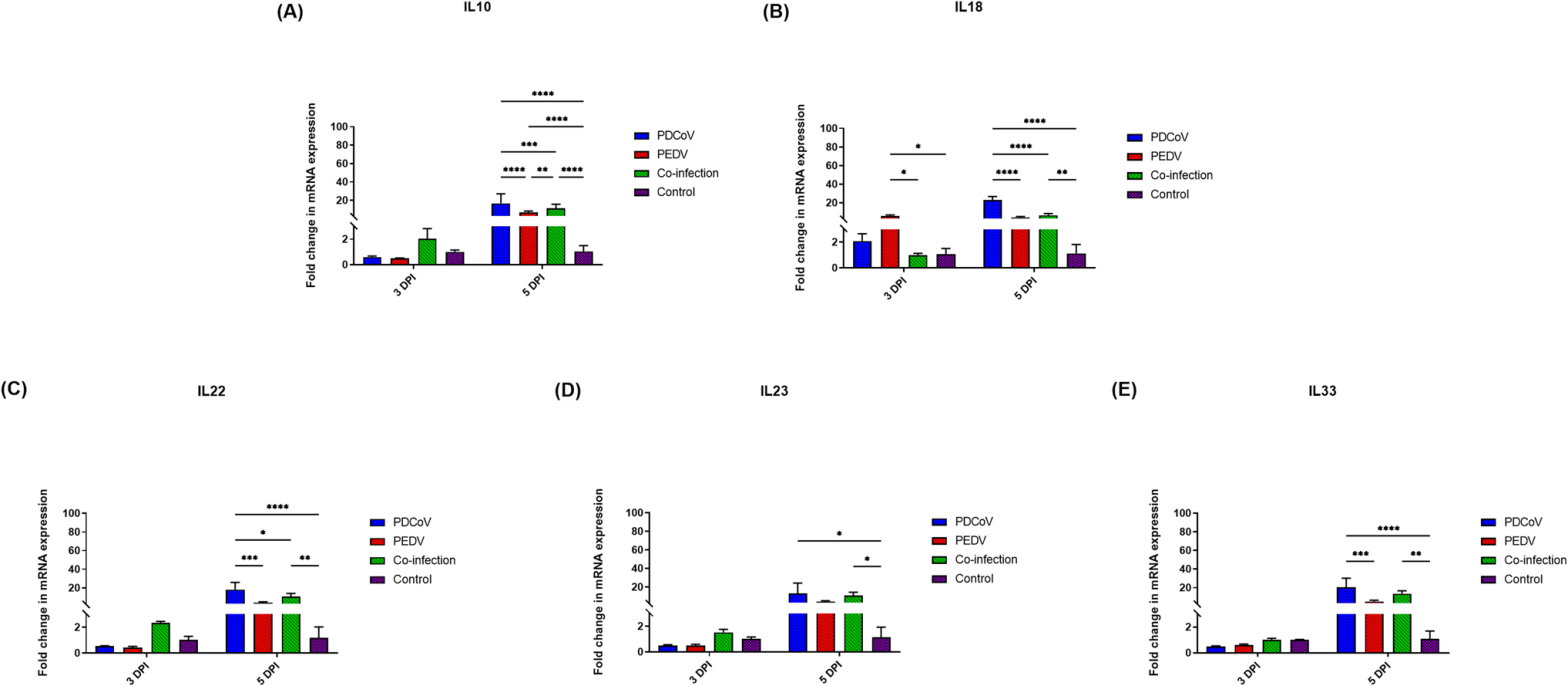
Messenger RNA (mRNA) expression of regulatory cytokines on the intestinal mucosa of neonatal pigs. (**A**) The mRNA levels of interleukin 10 (IL10). (**B**) The mRNA levels of interleukin 18 (IL18). (**C**) The mRNA levels of interleukin 22 (IL22). (**D**) The mRNA levels of interleukin 23 (IL23). (**E**) The mRNA levels of interleukin 33 (IL33). The mRNA levels were determined using qPCR. Glyceraldehyde-3-phosphate dehydrogenase (GAPDH) and beta-actin were used as an internal control to normalize changes in specific gene expressions. The results were presented as fold changes relative to the control animals. Different lower-case letters indicate significant differences between each group and each day post inoculation (DPI) (*p* < 0.05).

The expression levels of regulatory cytokine genes, including IL10, IL18, IL22, IL23, and IL33, are presented in Fig. 7. The PDCoV/ PEDV-co-inoculation group showed the significant up-regulation of IL10, IL18, IL22, IL23, and IL33 at 5 DPI as compared to the control group (IL10: 11.51-fold change; *p* < 0.0001; IL18: 6.60-fold change; *p* = 0.0052; IL22: 10.56-fold change; *p* = 0.0078; IL23: 10.96-fold change; *p* = 0.0453; IL33: 71.09-fold change; *p* = 0.0039) (Fig. 7). The single-PEDV-inoculated group showed the significant up-regulation of IL18 at 3 DPI as compared to the control group (5.72-fold change; *p* = 0.0173) (Fig. 7B). The single-PDCoV-inoculated group did not show a significant regulatory effect on either cytokine genes at 3 DPI as compared to the control group. However, the pigs in the single-PDCoV-inoculation group showed a significant increment in IL10, IL18, IL22, IL23, and IL33 expression at 5 DPI as compared to the control group (IL10: 16.80-fold change; *p* < 0.0001; IL18: 22.82-fold change; *p* < 0.0001; IL22: 18.13-fold change; *p* < 0.0001; IL23: 13.14-fold change; *p* = 0.0126; IL33: 20.51-fold change; *p* < 0.0001) (Fig. 7), while the single-PEDV inoculation group showed a significant up-regulatory effect only for the IL10 gene at 5 DPI as compared to the control group (7.02-fold change; *p* < 0.0001) (Fig. 7A).

## Discussion

**Figure 8:**
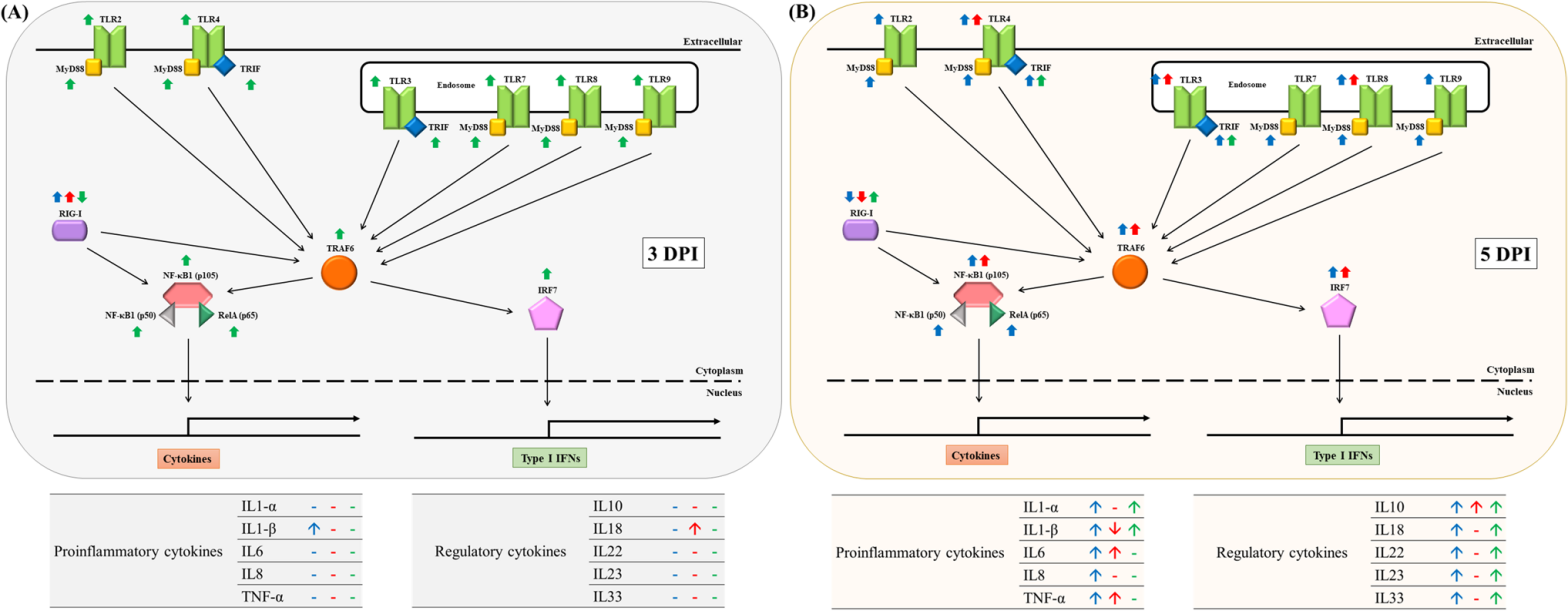
The modulations of pattern recognition receptors (PRRs), downstream mediators, and inflammatory cytokines in porcine deltacoronavirus (PDCoV) and/or porcine epidemic diarrhea virus (PEDV) infected neonatal piglets. (**A**) Gene modulation at 3-day post-inoculation (DPI). (**B**) Gene modulation at 5-day post-inoculation (DPI). Up and down arrows represent gene upregulation and down-regulation, respectively. Blue, red, and green arrows represent PDCoV single-inoculated, PEDV single-inoculated, and co-inoculated groups, respectively.

Porcine enteric coronavirus, including PDCoV and PEDV, produces significant enteric atrophy by targeting the epithelial cell of the intestinal villi. In addition, to enterocytes, intestinal immune cells, including microfold cells, dendritic cells, lymphocytes, and macrophages, are an important first line of defense and part of the innate intestinal immune system. Pattern recognition receptors (PRRs), including RIG-I-like receptor and TLRs, are constitutively expressed in innate immune cells and play a vital role in viral recognition, leading to antiviral signaling cascades^10–13^. Specifically, cell membranes (TLR2 and TLR4) and endosomal-associated (TLR3, TLR7/8, and TLR9) TLRs and cytosolic RIG-I like receptors play an important role in recognizing viral nucleic acids, while TLR2 and TLR4 have been demonstrated to recognize envelope glycoproteins^29–32^. Previous studies reported the modulatory effect of either single-PDCoV or single-PEDV infection on TLRs and RIG-I-like receptors, their downstream mediators, and cytokines’ mediated response in in vivo and in vitro models^12,17,33–36^. However, the modulatory effect of the genes involved in these immune mediated pathways during PDCoV/PEDV co-infection has not been thoroughly evaluated. Our results demonstrated that PDCoV/PEDV co-infection induces an earlier gene modulatory response on the part of the TLR/signaling pathways, which leads to the earlier modulation of cytokine-mediated genes as compared with a modulatory effect during PDCoV or PEDV single infection (Fig. 8).

Although TLR3 recognizes double-stranded RNA (dsRNA) viruses, it can be activated by the single-stranded RNA (ssRNA) of viruses such as West Nile virus (WNV) and the influenza A virus^37–39^. Similar to other coronaviruses, PDCoV and PEDV can produce dsRNA intermediates during replication, which may act as pathogen-associated molecular patterns (PAMPs) recognized by TLR3^40,41^. Previous studies have demonstrated that either single-PDCoV (CHN-GD-2016) or -PEDV S-INDEL, but not non-S-INDEL, infection significantly upregulated TLR3 by 3 DPI^12,17^. The results of this study support the existence of an early TLR3 regulatory effect induced by a single infection, while PDCoV/PEDV co-infection showed an earlier up-regulatory effect, followed by downregulation at 5 DPI. The mechanism of this earlier response observed during co-infection was not evaluated in this study; however, we hypothesize that PEDV/PDCoV co-infection may have a synergistic effect that led to the early up-regulation of TLR3 as compared with single infection. Although TLR9 recognizes double-stranded DNA (dsDNA)^42,43^, it could be modulated by ssRNA, similarly to TLR7 and TLR8. Our results also demonstrate that single-PDCoV infection up-regulates TLR8 and TLR9, while single-PEDV infection up-regulated TLR8 by the end of the study. However, no significant mRNA regulatory effect on endosomal TLR7 was observed in the single-PDCoV- or -PEDV-inoculated groups. Thus, the effects of PEDV infection on TLR7, TLR8, and TLR9 expressions levels differed from those described in previous reports^12^, in which neonatal piglets inoculated with PEDV S-INDEL showed the significant up-regulation of TLR7 at 3, in addition to the down-regulation of TLR9 at 7 DPI, while neonatal piglets inoculated with PEDV non-S-INDEL (USA/IN19338/2013) showed the down-regulation of TLR7, TLR8, and TLR9 by 3 DPI^12^. Interestingly, PDCoV/PEDV co-infection showed a transient up-regulatory effect on TLR7, TLR8, and TLR9 by 3 DPI. The earlier up-regulatory effect of PEDV/PDCoV co-infection in the expression levels of TLR7, TLR8, and TLR9, as compared with the single-infection groups, support our hypothesis concerning the potential synergistic effect. Previous studies have suggested that PEDV could enhance PDCoV replication in the intestinal villi and crypts, as well as exacerbating viral shedding during PEDV/PDCoV co-infection as compared with the single-PDCoV infection^18,44^. Perhaps these previous changes observed during coinfection are the result of molecular changes associated with an earlier regulation of TLRs in the PEDV/PDCoV co-infection group, as observed in this study.

Transmembrane PRRs, TLR2, and TLR4 can sense viral nucleic acid and viral structural and nonstructural proteins^32,45–47^. Both TLRs are reported to be modulated by PDCoV and PEDV^12,48^. Previous studies have shown that PEDV can affect TLR2 and TLR4 modulation in a subtype-dependent manner. Thus, PEDV S-INDEL, but not non-S-INDEL infection, can down-regulate TLR2^12^. Meanwhile, PDCoV has been reported to up-regulate TLR2 in swine testicular (ST) cells^48^. However, there is no previous information regarding the effect of PDCoV on TLR2 in vivo. In this study, PEDV inoculation with a non-S-INDEL isolate, P1915-NPF-071511A (99.99% amino acid identity with PEDV non-S-INDEL strain USA/IN19338/2013 based on S gene), showed similar results to non-S-INDEL in vivo inoculation reports, supporting the idea of the subtype-dependent regulation of TLR2. Meanwhile, single-PDCoV infection and PDCoV/PEDV co-infection up-regulate TLR2 by 5 DPI and 3 DPI, respectively. The differential modulatory effect of TLR4 has also been reported to be associated with various PEDV subtypes characterized by PEDV S-INDEL-induced upregulation and non-S-INDEL-induced downregulation^12^. Similar to TLR2, only in vitro information associated with the modulatory effect of PDCoV on TLR4 has been reported^48^. Previous studies have shown that non-S-INDEL induces down-regulation by 3 DPI, which is sustained at 7 DPI. Although, in this study, the down-regulatory effect observed by 3 DPI was not significant, there was a significant up-regulation by 5 DPI. Due to differences in the timeframes evaluated between our results and the previous results, it could be speculated that PEDV non-S-INDEL can exert an acute down-regulatory effect on TLR4 by 3 DPI, followed by a transient up-regulation by 5 DPI. In addition, the lack of significance for TLR4 modulation observed by 3 DPI may also be due to differences in experimental design, including infectious dose, between studies. Here, we observed that PDCoV/PEDV co-infection only has a detectable effect on TLR4 modulation by 3 DPI and single-PDCoV induces TLR4 upregulation by 5 DPI, similar to TLR2. Similarly, with the modulatory effect observed on endosomal-associated TLRs, the results observed on cell-membrane TLR2 and TLR4 supported the potential synergistic effects that result in enhanced PDCoV replication during PEDV co-infection^18,44^.

Most TLRs use a common signaling molecule adaptor, MyD88, to regulate type I IFN or inflammatory cytokine production via the activation of NF-kB^49^. The exception regarding this intricate regulatory effect is observed for TLR3, which mediates inflammatory response through TRIF, and TLR4, which can dually utilize MyD88 and TRIF^49^. Previously, PEDV non-S-INDEL was reported to down-regulate MyD88 and TRIF by 3 DPI, with no detectable effect by 7 DPI^12^. Conversely, in this study, single-PEDV infection did not significantly affect MyD88 or TRIF modulation. Although single-PEDV infection showed up-regulation on cell-membrane- (TLR4) and endosomal-associated (TLR3 and TLR8) TLRs by 5 DPI, it seems that these TLRs did exert a sufficient modulatory effect on MyD88 and TRIF. Similar to the results observed for TLR4, the absence of a detectable mRNA modulatory effect for MyD88 genes, as compared with previous reports in which single-PEDV infection induced an obvious modulatory effect on MyD88, may be the result of differences in the timeframe and infectious dose used across studies. No previous information regarding the effect of PDCoV on MyD88 or TRIF has been reported in in vitro or in vivo models. In this study, both MyD88 and TRIF were up-regulated by 5 DPI in single-PDCoV-infected pigs. The up-regulatory effect on MyD88 and TRIF may be the result of direct viral interaction or the up-regulation effect on cell-membrane- (TLR2 and TLR4) and endosomal-associated (TLR3, TLR8, and TLR9) TLRs induced by single-PDCoV infection. Also, PDCoV/PEDV co-infection has induced a higher up-regulatory effect on MyD88 and TRIF by 3 DPI as compared with single-PDCoV or PEDV infection. Similar to the modulatory effect observed for cell-membrane- (TLR2 and TLR4) and endosomal-associated (TLR3, TLR7, TLR8, and TLR9) TLRs, the results observed for MyD88 and TRIF support potential synergistic effects that result in enhanced PDCoV replication during PEDV co-infection^18,44^.

RIG-I recognizes short viral dsRNA, or ssRNA^50^, and PDCoV and PEDV have shown to exert different modulatory effects on these intracellular PPRs^51–55^. Previous studies have demonstrated that PDCoV N protein decreases RIG-I recognition and activation in HEK-293T cells^53^. Moreover, has been shown that RIG-I activity can be blocked in LLC-PK1 cells by PDCoV infection^54^. However, there is no previous information regarding the effect of PDCoV on RIG-I in vivo. The modulation of RIG-I in vivo has only been observed during S-INDEL infection but not non-S-INDEL infection^12^. In this study, both single-PDCoV and -PEDV-infection up-regulate RIG-I by 3 DPI, followed by downregulation by 5 DPI. The different effects on RIG-I modulatory effects induced by PEDV non-S-INDEL infection observed between the present and previous studies may be due not only to interaction with S protein but other structural proteins expressed during the acute phase of infection. Thus, PEDV E protein was reported as a down-regulatory modulatory protein on the RIG-I gene in porcine intestinal epithelial cell line-J2 (IPEC-J2)^56^. Further studies will be necessary to elucidate the specific role the different structural proteins of PEDV on RIG-I modulation in in vivo models. Conversely to single-PDCoV or -PEDV infection, PDCoV/PEDV co-infection showed a down-regulatory effect on RIG-I by 3 DPI, followed by up-regulation by 5 DPI. These results also support the idea that synergistic effects lead to the early down-regulation of these PRRs. The RIG-I receptor is an important viral RNA sensor that initiates a rapid innate immune response^57^. Immune evasion via the avoidance of RIG-I viral recognition is a common strategy used by RNA viruses^58^. Therefore, in this study, we hypothesize that PDCoV/PEDV co-infection enhances viral escape, abrogating the mucosal innate immune response due to the exacerbated down-regulatory effect of RIG-I during the acute phase (3 DPI) and resulting in the individual increment of viral shedding.

The activation of RIG-I and the TLR’s downstream adaptors, MyD88 and TRIF, lead to the recruitment of TRAF6, resulting in the activation of NF-κB and IRFs^14,15^. Previously, PEDV S-INDEL and non-S-INDEL were reported to have a transient down-regulatory effect on TRAF6 in the intestinal mucosa by 3 DPI but not a detectable effect by 7 DPI^12^. However, the role of TRAF6 during PDCoV has not been reported in either in vitro nor in vivo models. In this study, single-PDCoV and -PEDV infection induce TRAF6 up-regulation by 5 DPI, with significantly higher levels of expression being associated with PDCoV as compared with PEDV. Because no detectable modulatory effect on the part of MyD88 and TRIF were observed during single-PEDV infection, the up-regulation effect of TRAF6 may be the result of direct viral infection. Meanwhile, the larger up-regulatory effect of TRAF6 observed during single-PDCoV infection as compared with single-PEDV infection may be the result of 1) direct viral interaction and 2) an additive effect on the part of TRAF6 regulation induced by the signaling molecule adaptors MyD88 and TRIF, which were highly up-regulated during single-PDCoV infection. However, a differential regulatory effect on TRAF6 by various viral proteins or even viral nucleic acids during coronavirus infection has not been reported. Thus, viral infection directly regulates TRAF6 in theory and should be further investigated. The activation of TRAF6 by single-PDCoV and -PEDV infection resulted in the activation of NF-κB and IRF7. A previous in vivo study has reported that PEDV non-S-INDEL did not affect NF-κB1 (p105) but down-regulated NF-κB1 (p50) and RelA (p65) by 3 DPI and IRF7 by 7 DPI^12^. Meanwhile, PDCoV infection has been shown to increase the production of NF-kappa-B (p65) encoded by RelA in vivo^59^. No previous information regarding the effect of PDCoV on IRF7, NF-κB1 (p105), and NF-κB1 (p50) in vivo has been reported. In this study, the up-regulation induced by single-PEDV infection on the NF-κB1 (p50) precursor NF-κB1 (p105) did not show a significant effect on the gene activation of the downstream heterodimer NF-κB1 (p50)/RelA (p65). Similar to RIG-I, the different effects on canonical NF-κB and IRF7 induced by PEDV non-S-INDEL infection observed between the current and previous studies may be due not only to S protein but also other proteins, such as the Nsp1, papain-like protease 2 (PLP2), N, and M proteins expressed during the acute phase of PEDV infection^33,60–62^. However, the differential modulatory effects of the canonical NF-κB and IRF7 produced by genetic and structural differences in each viral protein have not been described. Additionally, previous reports have suggested that PEDV-dependent NF-κB activity was associated with viral dose and active replication^33^. In contrast, in this study, we observed that RelA (p65) and NF-κB1 (p50) and its precursor NF-κB1 (p105) were up-regulated in single-PDCoV and PDCoV/PEDV coinfection but not single-PEDV infection. In addition, IRF7 was also up-regulated by single-PDCoV and -PEDV infection by 5 DPI, while PDCoV/PEDV co-infection induced the same regulatory effect by 3 DPI. These findings suggest that single-PEDV infection induced the TRAF6-mediated IRF7 signaling pathway, while single-PDCoV and co-infection may induce both the TRAF6-mediated canonical NF-κB and IRF7 signaling pathways. The results regarding an earlier modulatory effect observed during PDCoV/PEDV co-infection as compared with single-PDCoV or -PEDV infection suggest that viral co-infection induced the TRAF6-mediated canonical NF-κB and IRF7 signaling pathways, adding to the potential synergistic effects that result in enhanced PDCoV replication during PEDV co-infection^18,44^.

The activation of TLRs and RIG-I mediates antiviral signaling cascades, leading to cytokine production^27,28^. Our previous study suggested that single-PEDV infection up-regulates IFN-α but not IL12, while single-PDCoV infection up-regulated IFN-α and IL12 by 5 DPI. The early up-regulation of IFN-α and IL12 induced by PDCoV/PEDV co-infection resulted in a sustained up-regulatory effect on IL12, which remained detectable until the end of study^18^. In this study, we additionally report the regulation of proinflammatory (IL1-α, IL1-β, IL6, IL8, IL18, and TNF-α) and regulatory (IL10, IL22, IL23, and IL33) cytokines associated with single-PDCoV and -PEDV infection, as well as PDCoV/PEDV co-infection. Previous studies reported that TNF-α modulation is PEDV strain dependent, and its infection does not have a detectable effect on IL6 gene modulation in the small intestine by 5 DPI^12^. However, additional studies have demonstrated that the regulatory effect of PEDV in the intestinal mucosa occurs much earlier, with the significant positive mRNA modulation of IL6 and other proinflammatory cytokines, including IL1-α, IL1-β, IL8, and IL18, which is detectable at 6 h post-infection (hpi)^63^. Although the duration of this early proinflammatory cytokines activation has not been previously described, in this study, we observed that, during single-PEDV infection, there is an up-regulatory effect on the part of IL18 that remains detectable by 3 DPI, while an opposite regulatory effect was observed on IL1-β, and no modulation of IL1-α or IL8 by 5 DPI was found. Other studies have suggested that PEDV S-INDEL enhances proinflammatory cytokine production through the non-canonical NF-κB pathway by stimulating RIG-I, while PEDV non-S-INDEL suppresses proinflammatory cytokine production through the canonical NF-κB pathway^12^. Because the TRAF6-mediated canonical NF-κB signaling pathway was not associated with single-PEDV infection in this study, we hypothesize that the regulatory effect of IL1β and IL18 could be the result of noncanonical NF-κB signaling pathway activation, led by RIG-I regulation. In contrast with previous reports, here, we observed the strong up-regulation of TNF-α and IL6 by 5 DPI. Although the p38 MAPK pathway was not evaluated in this study, previous studies suggest that it is possible that PEDV infection up-regulates TNF-α and IL6 through the TLR4-dependent p38 MAPK pathway^64–66^. Previously, it has been demonstrated that PDCoV can up-regulate IL1β and TNF-α but not IL6 in the intestinal mucosa after 5 DPI in 7-day-old pigs^59^. No previous studies have reported the regulation of IL8 and IL18 by PDCoV. Similarly, in this study, single-PDCoV infection up-regulated the proinflammatory cytokines IL1-β and TNF-α by 5 DPI; in addition, this study also showed PDCoV’s regulatory effect on a large number of proinflammatory cytokines, including IL1-α and IL6, as well as regulatory cytokines, including IL8 and IL18. Although further confirmation may be necessary to confirm the role of single-PDCoV infection, all the information generated in this study suggests that the pro- and regulatory cytokine effect during single-PDCoV infection could be modulated through the TRAF6-mediated canonical NF-κB signaling pathway and induce TLR activation. The differential regulatory effect on IL6 reported in this and previous studies could be due to the difference of viral strains and age of the infected pigs^67,68^. The regulatory effect of PDCoV/PEDV co-infection on proinflammatory cytokine response in mucosal immunity has not been previously reported. Interestingly, although PDCoV/PEDV co-infection showed an earlier up-regulatory effect on TRAF6-mediated canonical NF-κB and IRF7 signaling pathways by up-regulating TLRs as compared with single-PDCoV or -PEDV, only the up-regulation of the proinflammatory cytokines IL1-α, IL1-β, and IL18 was observed by 5 DPI. This result suggests that PDCoV/PEDV co-infection enhances viral escape, abrogating the mucosal innate immune response and resulting in an individual increase in viral shedding.

This study evaluated the regulatory effect of single- and PDCoV/PEDV co-infection on regulatory cytokines (IL10, IL22, IL23, and IL33). The upregulation of IL10 and IL22 mRNA induced by PEDV infection in the small intestinal mucosa was observed by 6 hpi^63^. The results of our study support a delayed up-regulatory effect induced by single-PEDV on IL10 by 5 DPI. In addition, single-PDCoV infection and PDCoV/PEDV co-infection showed a late modulatory effect, suggesting that PDCoV can also exert a positive regulatory effect on this regulatory cytokine. IL10 is considered an essential cytokine that inhibits the production of proinflammatory cytokines in several types of cells in the immune system^69^, and its stimulation could be a mechanism utilized by enteric coronaviruses to evade the mucosal immune response. Although PEDV has been linked with an early regulatory effect on IL22^63^, in this study, no effect was observed during single-PEDV infection. The differences in the time post-infection at which this IL22 was evaluated in these different studies are one of the main causes of this different result during PEDV infection. Meanwhile, single-PDCoV infection and PDCoV/PEDV co-infection were associated with the strong regulation of IL22 by 5 DPI, suggesting that PDCoV has a late regulatory effect on IL22. IL22 plays an important role in epithelial cell regeneration and tissue repairing^69^, and the combination of IL-10 and IL22 plays a vital role in mucosal damage prevention^70^. This mechanism, with the modulatory effect observed mostly during PDCoV infection, may support the less severe intestinal damage observed during PDCoV infection. There is no previous information regarding the effect of enteric coronavirus on IL23 and IL33. IL-23 functions in innate and adaptive immunity and is a key cytokine for promoting inflammatory responses in various target organs. The most important function ascribed to IL-23 is its role in the development and differentiation of effector Th17 cells via the activation of STAT3. In this study, single-PDCoV infection and PDCoV/PEDV co-infection but not on single-PEDV infection showed a late modulatory effect, suggesting that PDCoV but not PEDV has a primary regulatory effect on this regulatory cytokine, suggesting its role in more severe Th17 response. Further studies are necessary to evaluate the IL-23-associated pathway. Interleukin IL-33 is a new member of the IL-1 superfamily of cytokines, which is expressed mainly by stromal cells, such as epithelial and endothelial cells, and its expression is up-regulated following pro-inflammatory stimulation. IL-33 can function as a traditional cytokine and nuclear factor regulating gene transcription. In this case, a late modulatory effect was observed for single-PDCoV and PDCoV/PEDV co-infection but not single-PEDV single infection, suggesting the primary role of PDCoV for this regulatory cytokine in neonatal coronavirus infection. The up-regulatory effect of IL33 was consistent with the up-regulation of My88 and IL1 receptor; in this case, both are observed only in the presence of PDCoV, indicating that this regulatory interleukin could have a direct effect on the production of pro-inflammatory response during PDCoV but not PEDV infection.

## Conclusion

This study is the first report on the modulation of PRRs, downstream mediators, and cytokines in single-PDCoV-infected and PDCoV/PEDV-co-infected neonatal piglets. We suggest that single-PEDV regulates the noncanonical NF-κB signaling pathway through RIG-I regulation. Meanwhile, single-PDCoV infection and PDCoV/PEDV co-infection regulate proinflammatory and regulatory cytokines through TRAF6-mediated canonical NF-κB and IRF7 signaling pathways through TLRs. Although PDCoV/PEDV co-infection had an earlier modulatory effect on the signaling pathways, the regulation of proinflammatory and regulatory cytokines was observed during single viral infection. We hypothesized that PDCoV/PEDV co-infection may have a synergistic effect that enhances viral escape by abrogating the mucosal innate immune response. Although neonatal PDCoV/PEDV co-infection seems to have a differential modulatory effect of genes associated with PRRs, downstream mediators, and cytokines compared with PDCoV-single infection, further studies may be necessary to evaluate if these genes modulatory effects are also translated to similar changes on the different effector's proteins.

## Data Availability

The data that support the findings of this study are available on request from the corresponding author.
